# Gender diversity of editorial boards and gender differences in the peer review process at six journals of ecology and evolution

**DOI:** 10.1002/ece3.5794

**Published:** 2019-11-08

**Authors:** Charles W. Fox, Meghan A. Duffy, Daphne J. Fairbairn, Jennifer A. Meyer

**Affiliations:** ^1^ Department of Entomology University of Kentucky Lexington KY USA; ^2^ Department of Ecology & Evolutionary Biology University of Michigan Ann Arbor MI USA; ^3^ Department of Evolution, Ecology and Organismal Biology University of California Riverside CA USA; ^4^ British Ecological Society London UK

**Keywords:** bias, discrimination, editorial boards, equality, gender, peer review, scholarly publishing, women in science

## Abstract

Despite substantial progress for women in science, women remain underrepresented in many aspects of the scholarly publication process. We examined how the gender diversity of editors and reviewers changed over time for six journals in ecology and evolution (2003–2015 for four journals, 2007–2015 or 2009–2015 for the other two), and how several aspects of the peer review process differed between female and male editors and reviewers. We found that for five of the six journals, women were either absent or very poorly represented as handling editors at the beginning of our dataset. The representation of women increased gradually and consistently, with women making up 29% of the handling editors (averaged across journals) in 2015, similar to the representation of women as last authors on ecology papers (23% in 2015) but lower than the proportion of women among all authors (31%) and among members of the societies that own the journals (37%–40%). The proportion of women among reviewers has also gradually but consistently increased over time, reaching 27% by 2015. Female editors invited more female reviewers than did male editors, and this difference increased with age of the editor. Men and women who were invited to review did not differ in whether they responded to the review invitation, but, of those that responded, women were slightly more likely to agree to review. In contrast, women were less likely than men to accept invitations to serve on journal editorial boards. Our analyses indicate that there has been progress in the representation of women as reviewers and editors in ecology and evolutionary biology, but women are still underrepresented among the gatekeepers of scholarly publishing relative to their representation among researchers.

## Graphical abstract

We examined how the gender diversity of editors and reviewers changed over time for six journals in ecology and evolution, and how several aspects of the peer review process differed between female and male editors and reviewers. Our analyses indicate that there has been progress in the representation of women as reviewers and editors in ecology and evolutionary biology, but women are still underrepresented among the gatekeepers of scholarly publishing relative to their representation among researchers.

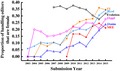

## 1 INTRODUCTION

The scholarly community has changed dramatically over the last century. One notable change is that women—who were once largely denied access to formal training in scholarly disciplines or relegated to uncredited or supporting roles (Wellenreuther & Otto, 2016)—now earn a sizeable proportion of graduate degrees (e.g., European Commission, 2015; National Science Foundation, 2015). Despite this progress, women continue to be underrepresented among recipients of science and engineering degrees, and remain even more underrepresented in academic leadership and other positions that determine the scientific agenda (Wellenreuther & Otto, 2016). This extends into the realm of scholarly publication. Women remain underrepresented among reviewers of journal papers (Fox, Burns, & Meyer, 2016a; Helmer, Schottdorf, Neef, & Battaglia, 2017; Lerback & Hanson, 2017). Women also remain underrepresented among the gatekeepers of scientific publishing; while representation varies substantially among disciplines and among journals within disciplines (Amrein, Langmann, Fahrleitner‐Pammer, Pieber, & Zollner‐Schwetz, 2011; Morton & Sonnad, 2007; Topaz & Sen, 2016), when compared to the gender of authors in a journal, women are underrepresented on editorial boards (Fox, Burns, & Meyer, 2016a; Helmer et al., 2017; Manlove & Belou, 2018; Topaz & Sen, 2016; Wehi, Beggs, & Anderson, 2019), especially at more senior editorial levels, for example, editors in chief (Amrein et al., 2011; Cho et al., 2014). While it is clear that women are underrepresented as reviewers and editors, we still lack a clear understanding of the causes and consequences of this gender disparity.

Low female representation on editorial boards can influence the research community in diverse ways. Appointment to an editorial board conveys a degree of prestige that may influence hiring, tenure, or promotion decisions by employers. Appointment to an editorial board also provides opportunities for intellectual growth and networking that can improve the quality of a research program and generate novel opportunities (Topaz & Sen, 2016). When editorial boards are male‐dominated, benefits such as these are disproportionately available to men. In addition, low diversity at senior editorial positions can negatively impact the proportion of women at junior editorial positions if the gender of the senior editors influences the recruitment of women to entry‐level editorial positions (Mauleón, Hillán, Moreno, Gómez, & Bordons, 2013). This can in turn impact the diversity of future senior editors (e.g., editors in chief) if senior editors are chosen from lower editorial ranks, creating a feedback loop maintaining high male representation on editorial boards.

Low gender diversity on journal editorial boards can also influence multiple aspects of scholarly publishing. Men and women can differ in their experiences and values (though there is tremendous variation within groups and overlap between them), which can influence their research interests and/or perspectives on scientific priorities. Differences in experiences between men and women might explain differences in perspectives toward the fairness of peer review (Bacchelli & Beller, 2017; Ho et al., 2013) and open access publishing (Alzahrani, 2010), perspectives that influence journal management decisions. Demographic diversity also promotes intellectual diversity, altering research trajectories even within subdisciplines (Stewart & Valian, 2018). For example, social status influences how people perceive others; however, it was only after women entered psychology in substantial numbers that studies considered how gender modulates that effect (Stewart & Valian, 2018). Given this, poor representation of women among the scientific gatekeepers is likely to reduce the diversity of ideas, perspectives, and values that make it to print: increased representation of women might change which types of manuscripts are accepted for publication, which areas are identified as worthy of invited reviews, which papers are selected to be highlighted by commentaries, and who is chosen to write those commentary and perspective pieces. Invited perspectives are disproportionately written by men (Baucom, Geraldes, & Rieseberg, 2019; Conley & Stadmark, 2012). Part of this may result from differences in the social and professional networks of men and women (McDonald, 2011; McPherson, Smith‐Lovin, & Cook, 2001), which likely influences who is selected to contribute invited papers or to review for the journal, especially when editors choose from among people they know or at least have interacted with. Men and women can also differ (on average) in the criteria they use when choosing prospective reviewers for peer review. For example, male editors generally consider reviewer status more highly during reviewer selection than do female editors (Grod, Lortie, & Budden, 2010), and some evidence suggests that male editors of ecology journals choose fewer women as reviewers than do female editors (Buckley, Sciligo, Adair, Case, & Monks, 2014; Fox, Burns, & Meyer, 2016a; Helmer et al., 2017; Lerback & Hanson, 2017) and that this difference varies with editor age (Fox, Burns, & Meyer, 2016a).

Being underrepresented in reviewer populations can influence the career development of scientists if, for example, reviewing provides positive benefits such as an opportunity to develop research evaluation skills or make positive impressions on editors (Lerback & Hanson, 2017), or if it leads to women being invited to serve on editorial boards. It is also important because it signals to the person who is asked to review that they are a respected member of their field (Lerback & Hanson, 2017), and because having fewer women reviewers can lead to fewer women writing perspective pieces, which shape the field and indicate a level of prominence for the author (Baucom et al., 2019). Gender differences in reviewer selection might also influence the peer review process if women review differently than do men. Women might have different views on the strengths and weaknesses of a study, and some studies suggest that female reviewers are more likely to recommend rejection than are male reviewers (Borsuk et al., 2009; Wing, Benner, Petersen, Newcomb, & Scott, 2010), though others do not observe this (Fox, Burns, & Meyer, 2016a; Fox, Burns, Muncy, & Meyer, 2016b and references therein).

Thus, there is clear evidence that women are underrepresented among editor and reviewer populations, and this likely influences both what gets published and the career progression of women. Despite that, we still do not fully understand the causes and consequences of female underrepresentation because few studies have examined how gender of editors or reviewers influences any particular aspect of the peer review process. In a previous study of one journal, *Functional Ecology*, Fox, Burns, and Meyer (2016a) observed that the gender and age of handling editors predicted the proportion of women invited to review for the journal (female editors invited more women to review, with the gender difference increasing with editor age) and the responses of those invitees to the review invitation (e.g., women were more likely to agree to review than were men). However, that study examined only a single journal and the degree to which those observations can be generalized is unclear.

In this study, we examine the gender diversity of editorial boards and its relationship with reviewer recruitment at six ecology and evolution journals—*Evolution*, *Functional Ecology*, *Journal of Animal Ecology*, *Journal of Applied Ecology*, *Journal of Ecology*, and *Methods in Ecology and Evolution*, all of which are highly ranked journals (e.g., all ranked in the top 25 by impact factor in the “ecology” category, with 2015 impact factors >4.0). We examine how the gender ratio of the editorial boards of these six journals has varied over time, test whether editor gender predicts the proportion of women that are invited and/or agree to review, examine how responses to review invitations differ between male and female invitees to review and examine how editor age mediates observed differences between male and female editors and reviewers. For a subset of the journals, we also look at gender differences in responses to invitations to join editorial boards.

## 2 METHODS

### 2.1 The peer review dataset

All six journals examined here use *ScholarOne Manuscripts* (previously *Manuscript Central*) to manage submissions and peer review. We extracted peer review data for all manuscripts submitted between 1 January 2003 and 30 June 2015 for *Functional Ecology*, *J Animal Ecology*, *J Applied Ecology,* and *J Ecology*, between August 13, 2009, and June 30, 2015, for *Methods in Ecology and Evolution* (this journal received its first ever submission on August 13, 2009), and between May 20, 2007, and December 31, 2015, for *Evolution* (*Evolution* began using *ScholarOne Manuscripts* to manage for submissions in May 2007). We included in our dataset only standard research papers (called a “Research Article” at *Methods in Ecology and Evolution*, an “Original Article” at *Evolution*, and a “Standard Paper” at the other journals); we excluded review papers, commentaries, perspectives, editorials, brief communications, and other types of papers not considered typical full‐length research manuscripts. We considered only the first submission of a paper; papers invited for revisions were excluded, even if sent for a second round of peer review. Resubmissions of papers following rejection were considered in our dataset if they got a new manuscript number and were sent for new peer review. Additional details about the dataset are described in Fox and Paine (2019).

Our dataset includes 133,431 reviewer names selected by editors as potential reviewers, for 40,420 standard research papers. Of these selected reviewers, 113,687 were invited to review and 54,912 agreed to review.

### 2.2 Variables in our dataset

For each manuscript that fits the criteria defined above, we have information on whether the paper was assigned to an associate editor, whether it was sent for peer review, the names of all reviewers selected as potential reviewers by that editor (if entered into *ScholarOne Manuscripts*), whether (and when) each selected reviewer was invited, whether (and when) they responded to the invitation, whether they agreed, and whether they actually submitted a review. Reviewers are recorded as having not responded to an invitation if either a “no response” was specifically recorded or if the reviewer is listed as invited but has no response recorded or review submitted; this differs slightly from Fox, Burns, and Meyer (2016a) who treated empty cells as unknown and did not analyze reviewer response rates pre‐2007 due to the large number of empty cells.

### 2.3 Inferring gender

We inferred binary genders for reviewers in our dataset. However, we acknowledge that gender is a spectrum and that people define their own gender identity; because of this, our inferences may have been inaccurate in some cases, and we discuss the potential for this to cause harm in the Discussion. We used a two‐step process. We first entered given names into an online database (https://genderize.io) that includes >200,000 unique names from 79 countries and 89 languages (as of November 2016). The database returns the most likely gender for each given name, along with a probability of the most common gender given that name (estimated from the known individuals included in the database). Genderize performs very well for names in western countries (Karimi, Wagner, Lemmerich, Jadidi, & Strohmaier, 2016), but includes few nonwestern names. For names that were not found in https://genderize.io, or that were found but had a probability <0.95, we used Internet searches to infer the gender of the individual. We searched for personal web pages or entries in online databases (such as profiles on Google Scholar, https://www.Mendeley.com, https://www.ResearchGate.com, Twitter, or Facebook) that included a photograph of the individual, or for news stories that made mention of the individual using gender‐specific pronouns such as “he” or”she”. We inferred gender for 132,602 of 133,449 reviewer entries in our dataset; the rest are of unknown gender and excluded from analyses of reviewer gender.

### 2.4 Editor seniority

We identified the year in which each editor obtained their PhD from their CVs or personal web pages, or by using online thesis archiving tools such as ProQuest's Dissertations & Theses, British Library EThOS, or similar sites for other countries. We were able to obtain exact dates for almost all past editors; for the rest, we estimated their PhD award date from their publication address history. We calculated *Editor Seniority* as the year of interest (the submission year of a manuscript they handled as editor) minus their PhD graduation year.

### 2.5 Statistical analyses

Most of the response variables examined here were binary; for example, gender [male/female] or invited/agreed to review [yes/no], and so were analyzed using logistic regression (SAS Proc Glimmix with dist = binomial). The only variable that was not binary is the time it took reviewers to respond to the review invitation, which was analyzed using general linear models (SAS Proc GLM). All analyses were of the form *DependentVariable* = *Year *+ *IndependentVariables* + *TwoWayInteractions*. Further details are described as necessary as results are presented.

Note that some of the specific parameter estimates presented here differ slightly from those presented for *Functional Ecology* in Fox, Burns, and Meyer (2016a) because the dataset used here is larger and has minor corrections throughout. The increase in data quality is small, and the change in parameter estimates is likewise very small. Thus, the current contribution augments, rather than supplants, Fox, Burns, and Meyer (2016a).

## 3 RESULTS

### 3.1 Gender diversity of journal editors

**Figure 1:**
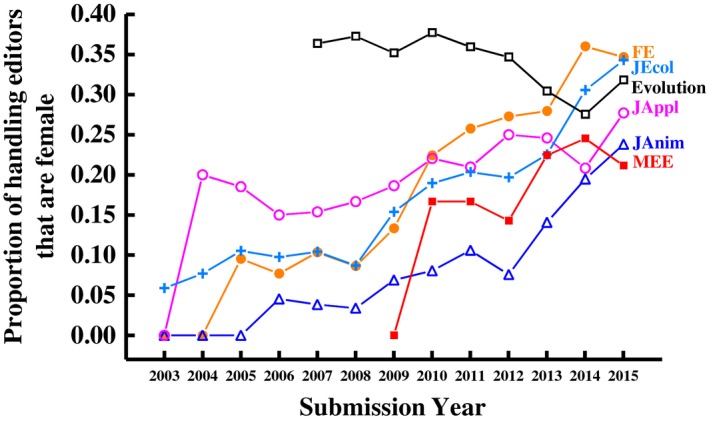
For five of the six journals, the proportion of handling editors that were female was very low at the start of the dataset, but improved over time. An editor was counted if they selected the reviewers for at least one manuscript that was submitted during the indicated year, irrespective of the number of papers they handled or their official appointment period (we do not have appointment dates for most editors)

For five of the six journals we studied, the proportion of women among editors was very small at the beginning of our dataset, and gradually but consistently increased over time (Figure 1). In 2003 and 2004, almost all editors handling manuscripts for *Functional Ecology*, *J Animal Ecology*, and *J Applied Ecology* were male (Figure 1). These journals were each edited by a small team of editors (three or four people at a time), none of whom were female—each had an “editorial review board” on which some women served, but these boards advised editors and occasionally reviewed papers but did not handle papers as editors. However, these journals switched editorial models in 2005, 2006, and 2004, respectively, to one in which Associate Editors choose reviewers for peer review and make decision recommendations to senior editors. Women were recruited as Associate Editors from the start of these editorial boards, but the boards were nonetheless very male‐dominated in the early years. *J Ecology*, in contrast, had a board of Associate Editors that predates 2003 and had some (although few) women handling manuscripts from the start of our dataset. *Methods in Ecology and Evolution* first received submissions in late 2009, with the first female editors handling manuscripts for the journal the following year.

The journal *Evolution* has operated under an editorial board model since its first issue in 1947 and had >35% female editorial board members in the earliest years (2007–2011) of our dataset, though this dipped below 30% in 2014.

Women made up <35% of the individuals handling reviewer selection and decision recommendations at all of these journals in 2015, the most recent year in our dataset. At three of the journals, <30% of the handling editors in 2015 were women (Figure 1).

### 3.2 Gender diversity of reviewers

**Figure 2:**
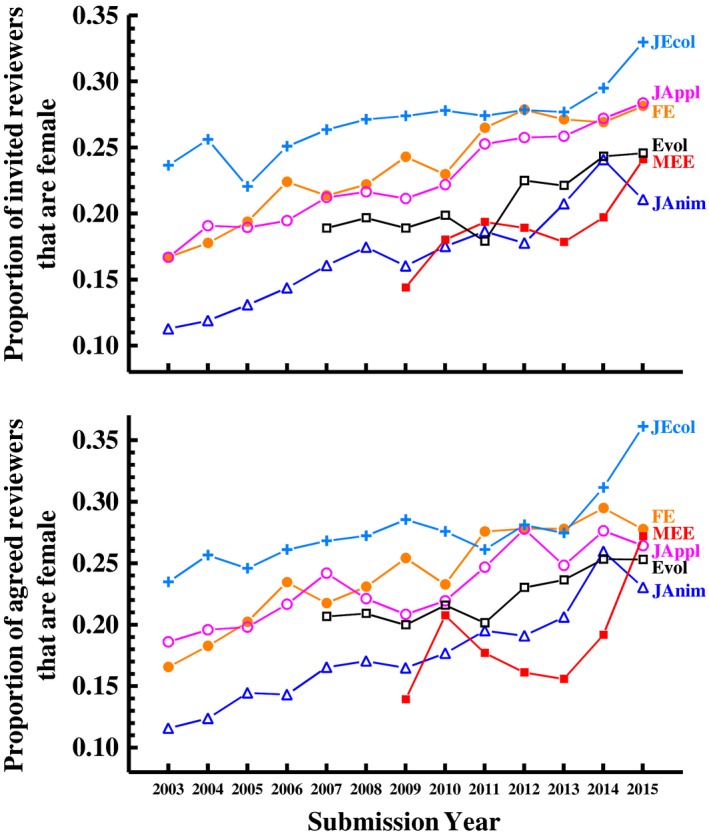
The proportion of invited reviewers that are women has been steadily increasing over time for six ecology and evolution journals. The mean sex ratio of invited reviewers varies among journals, but the rate of increase over time is similar among journals. This figure includes only individuals of known gender. Note that the specific parameter estimates presented for *Functional Ecology* here and in subsequent figures differ (though only *slightly*) from those presented in Fox, Burns, and Meyer (2016a) due to improved genderization of the data and further error correction that was done between that study and this one

The proportion of women among invitees to review varied among the six journals, but was low (<25%) for all of the journals in the first year the journal is present in our dataset (Figure 2a). This was true even at *Evolution*, which had the highest proportion of female handling editors until recently. The low proportion of women among invited reviewers translates into low proportions of women among the agreed reviewers (Figure 2b). However, the gender ratio of invited and agreed reviewers has been slowly but fairly consistently increasing over time at all of the journals, such that between 21% and 33% of all invited reviewers (Figure 2a) and between 23% and 36% of all agreed reviewers (Figure 2b) were female by 2015.

**Figure 3:**
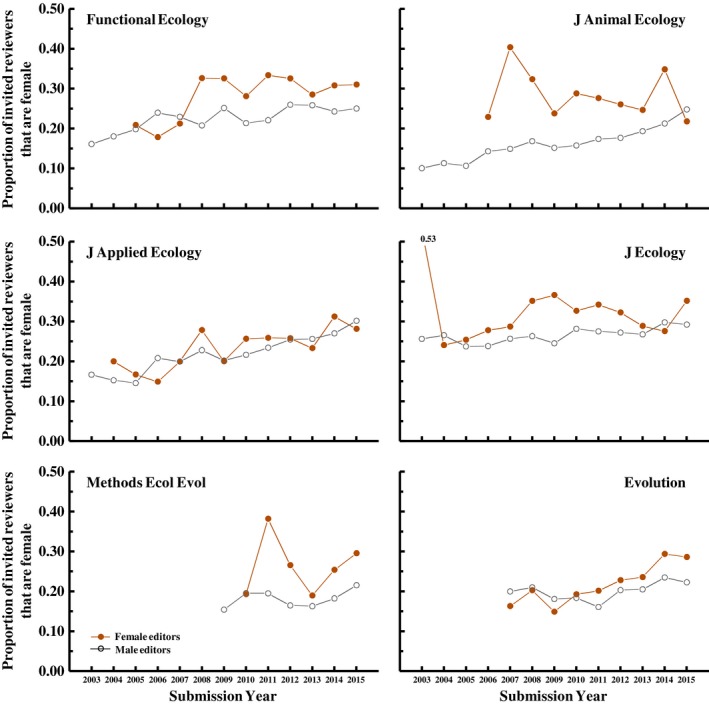
Female editors invite more women to review than do male editors at five of the six journals in our dataset (all except the *Journal of Applied Ecology*). Model: *ReviewerGender* [f/m] = Year + *Journal *+ EditorGender + 2‐way interactions, with *HandlingEditorID* as a random effect; *EditorGender*: $\chi_{1}^{2}$ = 22.3, *p* < .001; *Journal* * *EditorGender*: $\chi_{5}^{2}$ = 12.2, *p* = .03). Note that, the higher variance in estimates for female editors, especially in the earlier years, is because there were few female editors handling papers and so sampling error was high (e.g., only 1 of 18 handling editors was female for *J. Ecology* in 2003)

In a previous study, Fox, Burns, and Meyer (2016a) found that female editors of *Functional Ecology* invited more women to review than did male editors of that journal. Here, we see that this pattern is general—female editors, on average, invite 1.27 times as many women to be reviewers as do male editors (averaged across journals and years; Figure 3). However, this difference varies among journals (significant *Journal* * *EditorGender* interaction; Figure 3). In separate analyses for each journal (model: *ReviewerGender *[f/m] = Year + *EditorGender *+ interaction, with *HandlingEditorID* as a random effect), we see that female editors include more women among their invited reviewers at all journals except *J Applied Ecology* (*EditorGender* effect: $\chi_{1}^{2}$ > 4.9*, p* < .03 for all except *J Applied Ecology*, for which $\chi_{1}^{2}$ = 0.00, *p* = .99).

**Figure 4:**
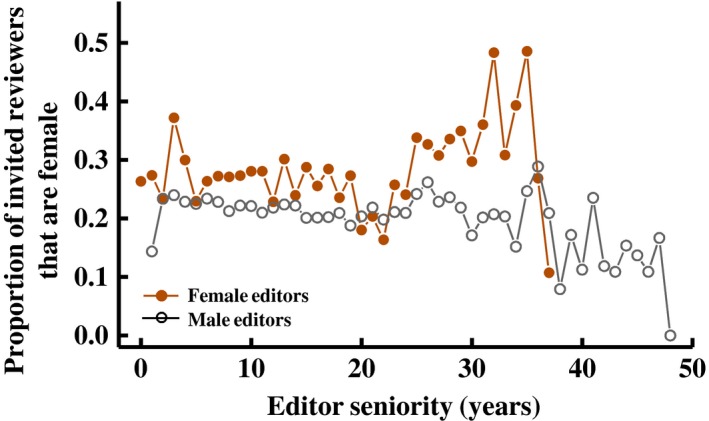
The proportion of women among invited reviewers varies with editor seniority (years since PhD), but this variation is different for men and women. On average, more senior women invite more women reviewers, but more senior men invite fewer women reviewers. Values presented in the figure are averages, first averaging across editors within each journal*year combination, then across years within each journal, and then across journals

The previous study by Fox, Burns, and Meyer (2016a) also found that the seniority of the handling editor (defined as years post‐PhD) influenced the proportion of women invited as reviewers, but that this effect differed between male and female editors—more senior female editors included a *higher* proportion of women among their invited reviewers compared to less senior female editors, whereas more senior male editors included a *lower* proportion of women among their invited reviewers than did younger male editors. Here, we find that this observation holds up when considering multiple journals (Figure 4)—the proportion of women among invited reviewers changed with editor seniority differently for male and female editors (model: *ReviewerGender *[f/m] = Journal + *Year *+ EditorGender + *EditorSeniority *+ 2‐way interactions, with *EditorSeniority* treated as a covariate; *EditorGender* * *EditorSeniority* interaction: $\chi_{1}^{2}$ = 9.9, *p* = .002). Specifically, the proportion of women among invitees to review increased with seniority for female editors (*t*_20,448_ = 3.67, *p* < .001) but did not change significantly with seniority for male editors (though the slope was negative; *t*_90,674_ = −1.58, *p* = .11; Figure 4), such that the difference in the proportion of women invited by female and male editors increased with editor seniority. However, the large difference between senior male and senior female editors does not account for all of the difference in the proportion of women invited by male and female editors; if we constrain our dataset to include only younger editors, the gender difference (women invite more female reviewers) persists for all age categories (editors <20 years seniority, *p* < .001; <15 years, *p* < .001; <10 years, *p* = .01).

### 3.3 Reviewer responses to review invitations

**Figure A1:**
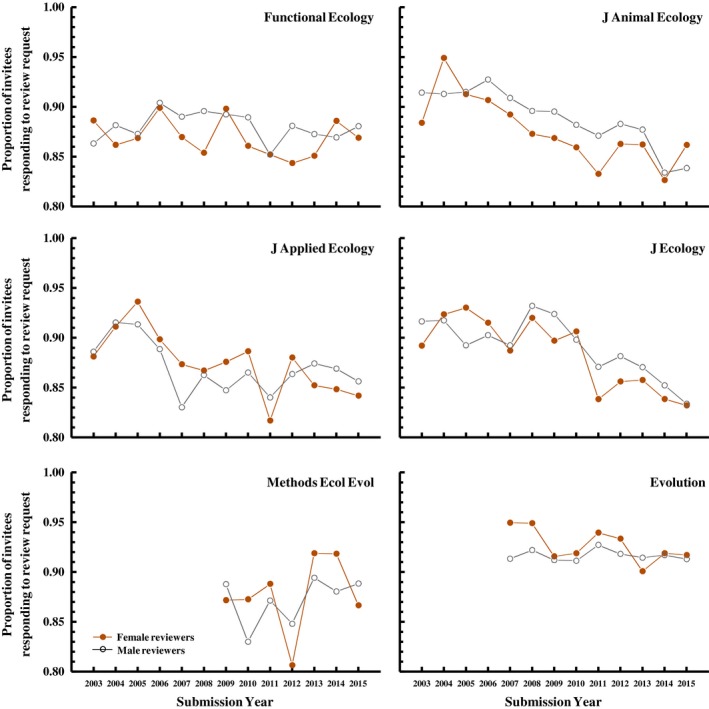
The proportion of invited reviewers that respond to review invitations for the five ecology journals published by the British Ecological Society, plus *Evolution*. On average, women are less likely to respond to the invitation to review, but the difference is small and varies across journals. Model: *ReviewerRespond*[y/n] = *Year* + *Journal* + *ReviewerGender* + 2‐way interactions; *Year*: ***x*** ^2^_12_ = 174.0, *p* < .001; *Journal*: ***x*** ^2^_5_ = 244.5, *p* < .001; *ReviewerGender*: ***x*** ^2^_1_ = 0.39, *p* = .53; Year*Journal: ***x*** ^2^_50_ = 263.3, *p* < .001; Year*ReviewerGender: ***x*** ^2^_12_ = 14.5, *p* = .27; Journal*ReviewerGender: ***x*** ^2^_5_ = 18.7, *p* = .002)

The proportion of reviewers responding to a review invitation (i.e., either by email or by clicking the link provided in the emailed invitation), and agreeing to review if they respond, varied among journals and over time (details in Fox, 2017; Fox, Albert, & Vines, 2017a). On average across all journals, we see no evidence that reviewer gender predicts how likely an invitee is to respond to the review invitation (Figure A1).

**Figure 5:**
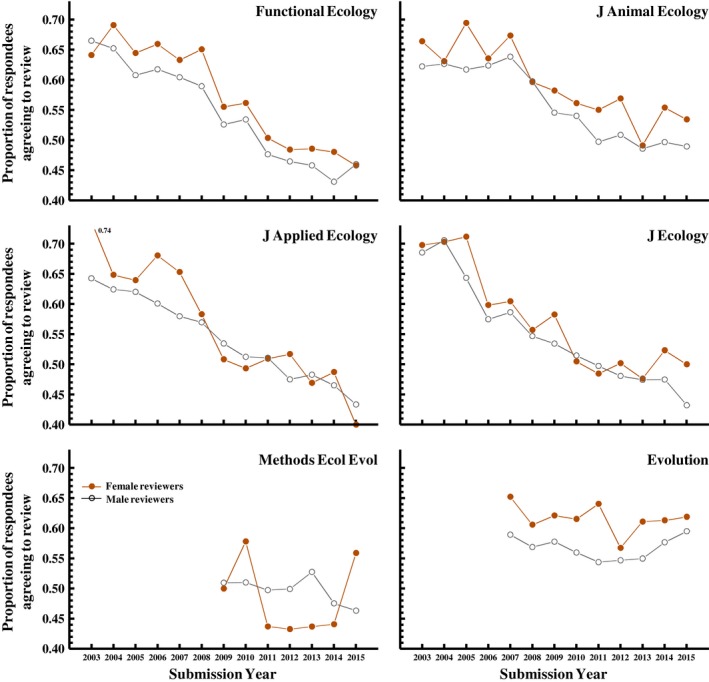
Women agree to review more often than do men, though the difference is small and there is substantial variation across years and among journals in the magnitude of this difference. This figure shows the proportion of male versus female respondents that agreed to review for the five journals published by the British Ecological Society, plus *Evolution*; see Figure A1 for data on the likelihood of responding to the invitation email. Model: *ReviewerAgreed *[y/n] = Year + *Journal *+ ReviewerGender + 2‐way interactions; *Year*: $\chi_{1}^{2}$ = 870.6, *p* < .001; *Journal*: $\chi_{5}^{2}$ = 166.2, *p* < .001; *ReviewerGender*: $\chi_{1}^{2}$ = 28.1, *p* < .001; *Year* * *Journal*: $\chi_{5}^{2}$ = 173.1, *p* < .001; *Year* * *ReviewerGender*: $\chi_{1}^{2}$ = 9.3, *p* = .67; *Journal* * *ReviewerGender*: $\chi_{5}^{2}$ = 15.6, *p* = .006)

Women that responded to the email invitation were more likely to agree to review than were men that responded to the email invitation (Figure 5), such that the overall representation of women among agreed reviewers was higher than their representation among invited reviewers. As with other variables examined, we see a significant *Journal* * *ReviewerGender* interaction (Figure 5) but, in separate analyses for each journal, the gender difference is statistically significant (at *p* < .02) for all except *Methods in Ecology and Evolution* (for which *p* = .46). For the five journals for which we see a difference, women agree to review on average 58.4% of the time (averaged across years within journals and then across journals) whereas men agreed just 55.3% of the time, an absolute difference of only 3.1%, but a *relative* increase in the proportion agreeing to review of 5.5% (or a relative decrease in the proportion declining to review of 7.0%).

Averaged across years and journals, 94.4% of agreed reviewers submitted a review to the journal. This number varied slightly across journals ($\chi_{1}^{2}$ = 30.0, *p* < .001; range: 92.8%–95.5%) and over time (though not consistent in direction; $\chi_{1}^{2}$ = 39.0, *p* < .001) but not between male and female reviewers (review submission rate for male and female reviewers, averaged across years and journals, was 94.3% and 95.0%, respectively, $\chi_{1}^{2}$ = 2.96, *p* = .09).

### 3.4 Does editor gender or age predict reviewer recruitment?

**Figure 6:**
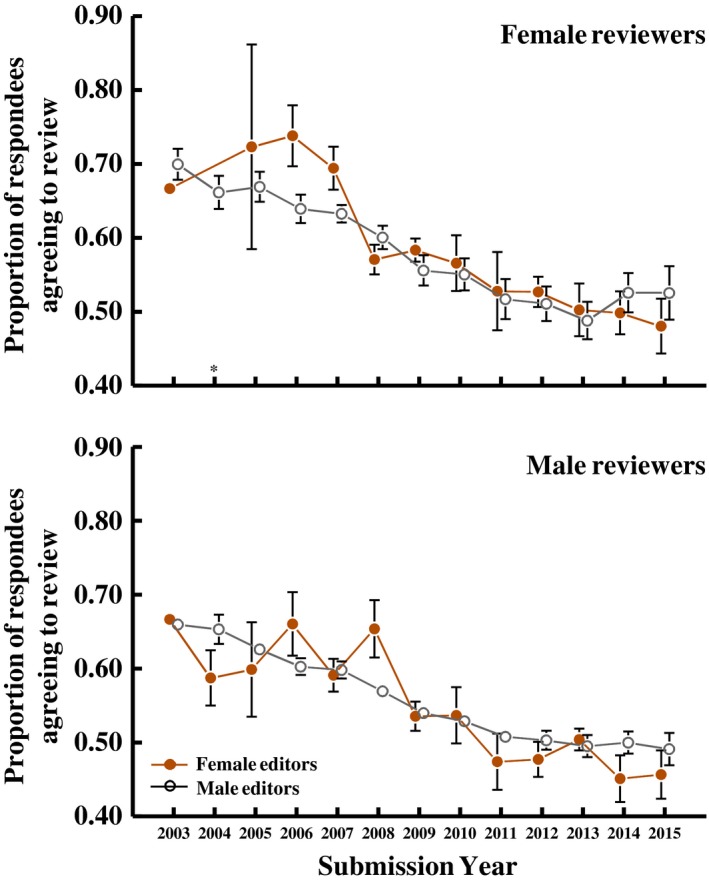
Averaged across all six journals, handling editor gender did not influence the likelihood that the respondent would agree to review. Means (±*SEM*) are averages across journals. Sample sizes for female editors are small in the earlier years. Note that, the EditorGender * *ReviewerGender* interaction is significant in a logistic regression (Model: *Respond *[y/n] = Year + *Journal *+ EditorGender + *ReviewerGender *+ 2‐way interactions; EditorGender * *ReviewerGender*, $\chi_{1}^{2}$ = 5.14, *p* = .02) but separate analyses for male and female reviewers (Model: *Respond *[y/n] = Year + *Journal *+ EditorGender + 2‐way interactions) fail to detect a significant influence of *EditorGender* on responses of either male reviewers ($\chi_{1}^{2}$ = 1.03, *p* = .31) or female reviewers ($\chi_{1}^{2}$ = 0.11*, p* = .74). All analyses include *HandlingEditorID* as a random effect

**Figure A2:**
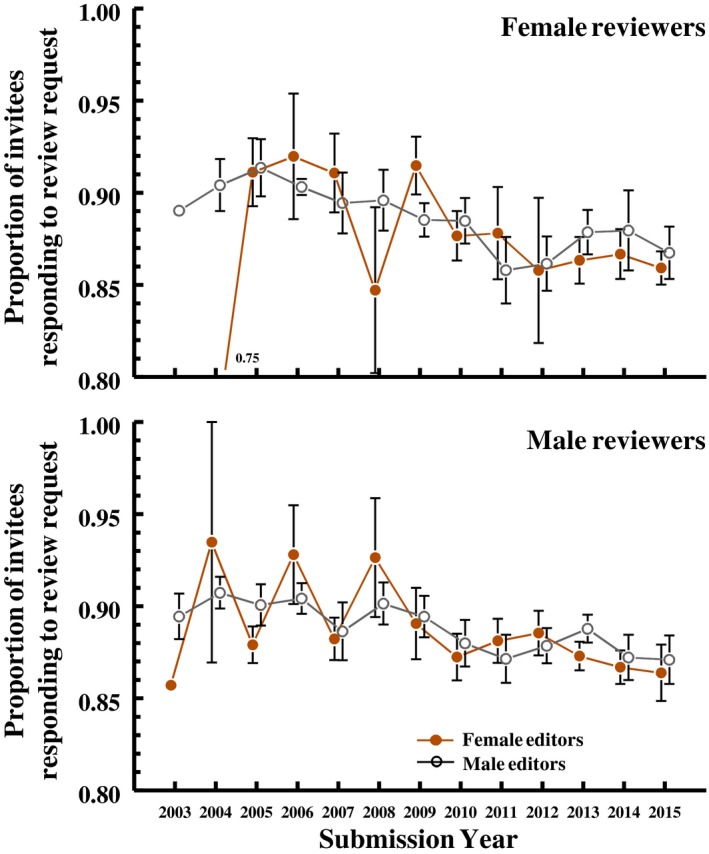
The proportion of invitees responding to the request to review when that request comes from a male versus female handling editor. Proportions are averaged across journals within years, and presented as mean ± SEM. Sample sizes for female editors are low in the earlier years. Model: *ReviewerAgreed*[y/n] = *Year* + *Journal* + *ReviewerGender* + *EditorGender* + 2‐way interactions; *ReviewerGender*: ***x*** ^2^_1_ = 0.58, *p* = .45; *EditorGender*: ***x*** ^2^_1_ = 2.04, *p* = .15; EditorGender*ReviewerGender: ***x*** ^2^_1_ = 0.26, *p* = .61)

In a previous analysis of *Functional Ecology* review invitations, Fox, Burns, and Meyer (2016a) observed that male invitees to review were slightly (but statistically significantly) less likely to respond to the review invitation and slightly less likely to agree if they responded, when the inviting editor was female rather than male. Female invitees to review did not respond differently to male versus female editors. However, when we consider all six journals we see little evidence that this gender difference is general; averaged across journals, reviewers were *not* more likely to respond to review requests from male editors, regardless of reviewer gender (Figure A2), nor were they more likely to agree to review if the editor was male (Figure 6; statistics in figure legends). When we evaluate individual journals, there was no individual journal for which invitees to review were more likely to respond to the review invitation when the editor was of their same gender (*ReviewerGender*‐×‐*EditorGender* interaction; $\chi_{1}^{2}$ < 0.55, *p* > .46 for all journals). The proportion of respondents (those that responded to the review invitation) that agreed to review was higher when the editor was the same sex as the reviewer at *Functional Ecology* (as previously reported by Fox, Burns, & Meyer, 2016a), but this was not the case at the other journals ($\chi_{1}^{2}$ < 0.22, *p* > .64 for all except one journal); for *J Appl Ecol,* reviewers of both genders were more likely to agree to review when the editor was male but the effect size differed between male and female reviewers ($\chi_{1}^{2}$ = 4.05, *p* = .044).

It was observed previously for *Functional Ecology* (Fox, Burns, & Meyer, 2016a) that more senior (i.e., older) editors had greater difficulty recruiting reviewers compared with younger editors. In our expanded dataset of six journals, we do not find that the proportion of invitees that responded to email invitations ($\chi_{1}^{2}$ = 8.3, *p* = .53) or the proportion of respondents that agreed to review ($\chi_{1}^{2}$ = 2.6, *p* = .11) varied with editor seniority (full model: *ReviewerResponse *[y/n] = Journal + *Year *+ EditorGender + *ReviewerGender* +EditorSeniority + 2‐way interactions, with *EditorSeniority* treated as a covariate). There was a significant *Journal* * *EditorSeniority* interaction for the proportion that agreed if responded ($\chi_{1}^{2}$ = 22.9, *p* < .001) but, in separate analyses for each journal, the editor seniority effect was statistically significant for only one journal ($\chi_{1}^{2}$ = 5.4, *p* = .02; *p* > .12 for the rest).

### 3.5 Recruiting editors

In contrast to the observation that women were more likely to agree to review than were men (see above), women were *less* likely to agree to join journal editorial boards than were men (model *Response *[y/n] = Journal + *Gender*; *Gender*: $\chi_{1}^{2}$ = 4.4, *p* = .04). At *J Ecology*, 92% of men invited to join their editorial board as an Associate Editor agreed whereas only 83% of women agreed (2012 to early 2016; *n* = 47). At *Functional Ecology*, 76% of men accepted the invitation whereas only 69% of invited women accepted (2005–2016, but includes only invitations sent by C. Fox; *n* = 205). At *Evolution*, 62% of invited men but only 52% of women agreed (2006–2015, spanning three different editors in chief; *n* = 316). None of these differences are large, but they are consistent in direction—women are 9%–16% (relative probability) less likely to join journal editorial boards of these journals when invited. Unfortunately, data are not available for the other three journals, nor for years outside those indicated above, due to differences in journal and editor record keeping procedures.

In 2017, the British Ecological Society (BES) published an “Open Associate Editor Recruitment” to recruit new Associate Editors for its five journals. The recruitment was advertised at many ecological conferences (including conferences in multiple countries in Europe, the United States, Mexico, Colombia, and China), through mailings to society membership and subscribers to journal tables of contents, on a variety social media platforms (including using the hashtags #womeninSTEM and #womeninscience), and on the Society's website. In total, 351 people from 48 countries applied to join one of the journals as an Associate Editor. Averaged across journals, just 27.2% of applicants were women (range across the five BES journals: 14.3%–47.6%). 36.3% of the new Associate Editor appointees were women (range: 31.3%–40.0%).

Similar gender distributions have been observed for BES Senior Editor recruitment. Between 2014 and late 2017, the BES advertised seven times for new Senior Editors. Between 0% and 57% of applicants for these Senior Editor, positions were women (average = 26.8%), and three of the seven new Senior Editor appointments were women.

## 4 DISCUSSION

Women have historically been underrepresented among editors and reviewers in scholarly journals. In this study, we examined (a) the gender diversity of the editorial and reviewer populations for six high impact factor journals in ecology and evolution and (b) how gender of editors and reviewers relates to several aspects of the peer review process. Our key results are (1) the proportion of women among journal editors was historically very low for five of the six journals examined (all except *Evolution*), but has gradually and consistently increased at these five journals such that women made up 21%–35% of the editors that chose reviewers for these journals in 2015; (2) the proportion of women among reviewers has also gradually but fairly consistently increased over time, with women comprising only 17% (averaged across journals) of invited reviewers in 2003 but 27% by 2015; (3) female editors include approximately 1.3 times as many women among their invited reviewers compared to male editors, but this difference varies with the age of the editor (it is larger for older editors) and among journals; (4) there was no gender difference in the proportion of invitees to review that responded to the invitation but, of those that responded, women were slightly more likely to agree to review; and (5) women are less likely to accept invitations to serve on journal editorial boards than are men.

### 4.1 Gender diversity of editorial boards

Despite being well‐represented among recipients of graduate degrees in the sciences, women are underrepresented on editorial boards relative to their frequency among authorships in the equivalent discipline throughout much of scholarly publishing (Cho et al., 2014, Helmer et al., 2017; Ioannidou & Rosania, 2015; Topaz & Sen, 2016). This underrepresentation was particularly substantial on the early editorial boards for five of the six journals examined here (all except *Evolution*). However, the representation of women has been steadily improving at these journals, with women representing ~29% of Associate Editors (averaged across journals) at these six journals as of 2015. The increase in the representation of women on editorial boards seen here is similar to that observed for other journals in ecology (data at the Gatekeepers Project; http://brunalab.org/gatekeepers), most of which have ~20%–40% female editors as of 2015.

It is unclear what specific proportion of women is expected on editorial boards to reflect their representation in the ecology and/or evolution communities. Though women currently obtain graduate degrees in the life sciences in similar numbers as men, this has not always been the case (Ceci, Ginther, Kahn, & Williams, 2014). This change in the number of women getting graduate degrees, and that women also are more likely to leave science than are men (Adamo, 2013; Goulden, Mason, & Frasch, 2011; Stewart & Valian, 2018), lead the representation of women to differ substantially between older versus younger scientists (Débarre, Rode, & Ugelvig, 2018; Martin, 2012; Stewart & Valian, 2018). But we can at least speculate on gender ratios that set reasonable targets. For example, women represented 34% of all authors of papers published in *Functional Ecology* in 2014 (averaged across all positions; Fox, Burns, Muncy, et al., 2016b), nearly the same as the proportion of women on the editorial board of this journal as of 2014–2015 (35%–36%). Across the broader ecology literature, women were ~31% of all authors between 2010 and 2015 (Fox, Ritchey, & Paine, 2018). However, women were only ~23% of last authors on papers during this same period (Fox et al., 2018); last authors are commonly the “senior” author, that is, the principal investigator or research supervisor (Duffy, 2017), which may better reflect the pool of people from which new editors are being selected. Indeed, 23% is close to the proportion of women that applied for a senior editor position at one of the British Ecological Society (BES) journals between 2004 and 2007 (27%) or responded to the BES's open call for new Associate Editors (also 27%). However, these gender ratios are substantially lower than the proportion of women in the broader ecological community. For example, the membership of British Ecological Society, which owns five of the journals examined here, was 39.9% women in 2014 (http://www.britishecologicalsociety.org/making-ecology-for-all-part-2), and the membership of the comparable North American society, the Ecological Society of America, was 37% as of 2010 (Beck, Boersma, Tysor, & Middendorf, 2014). In 2016, 40% of all members of the Society for the Study of Evolution (which publishes *Evolution*) were women, but only 33% of nonstudent members were women (Débarre et al., 2018), very close to the proportion of editors that handled papers for *Evolution* in 2015. Representation of women that fairly reflects the broader community of people qualified to be editors likely falls somewhere inside this broad range of gender ratios.

The representation of women on journal editorial boards varied quite substantially among the six journals examined here (a 13 percentage point difference from high to low in 2015). Most strikingly, we see that women have been well‐represented (at least compared with the other journals) for many years at *Evolution*, whereas equivalent female representation has only recently been achieved at the other journals. Even within the five journals published by the British Ecological Society, there is substantial variation in the gender ratios of their editorial boards. Interestingly, this variation reflects, at least roughly, similar variation among the specialties of ecology in the frequency of women as authors. For example, women are better represented as authors among most plant ecology subdisciplines, and among conservation biologists, than they are among vertebrate ecologists, mathematical ecologists, or statisticians (http://www.eigenfactor.org), concordant with the pattern of variation among journals that target these various communities. Given the variation in the proportion of women in various subcommunities of ecology and evolution, we should be cautious before passing judgment on the variation among journals in representation of women on their editorial boards. It would be particularly interesting to examine the factors that contribute to the underrepresentation of women in some subdisciplines.

Our data suggest that women are less likely than men to accept invitations to serve on editorial boards. Though our data were limited to just three journals—*Functional Ecology*, *Journal of Ecology* and *Evolution*—and limited to invitations sent by just five editors in chief, we nonetheless consistently observed that women were more likely than men to decline invitations to join editorial boards. It thus requires, on average across journals, invitations to ~1.5 women to recruit one new female editor, but only invitations to 1.3 men to recruit one new male editor. Though not a large difference, if equal numbers of men and women are invited to join a board, the observed difference in acceptance rate would lead to the proportion of men on the board exceeding women by ~seven percentage points.

We suspect that women are more likely to decline editor invitations because they have a greater number of other commitments and responsibilities than do men. There is a large body of evidence indicating that female scientists, especially those who have families, have greater demands on their time than do male scientists (Ledin, Bornmann, Gannon, & Wallon, 2007). Explanations provided in emails declining editor invitations suggest large differences in the types of commitments that lead men and women to decline an invitation. Of 50 emails declining the invitation to join the *Functional Ecology* editorial board (those still retained by C. Fox), 67% of men but only 38% of women invoked other editorial responsibilities as a major reason for declining the invitation (and 21% of men but only 4% of women mentioned the need for a break from previous editorial responsibilities), whereas 71% of women but only 21% of men referenced other noneditorial responsibilities that limited their time available to work as an editor (two women but no men specifically mentioned nonwork responsibilities; five people provided more than one explanation, and thus the totals add up to more than 100%). These differences may reflect how men and women describe their commitments, but they are also consistent with the common narrative that women have more personal and/or professional demands on their time other than working as an editor (Stewart & Valian, 2018).

### 4.2 Gender diversity of reviewers

As with editors, the proportion of women among individuals invited to review for these six journals has been steadily increasing over time. Interestingly, as of 2015 women are nearly equally represented among reviewers as they are among editors—27% versus 29%, respectively (averaged across journals). As discussed above, it is not clear what proportion of women among reviewers would reflect representation equal to that of women in the ecological community. However, given that the pool of reviewers tends to include more early career scientists (as compared to editors), and that women are better represented among early career ecologists (Stewart & Valian, 2018), we would expect greater representation of women among reviewers than editors.

Female editors include more women among their invited reviewers than do male editors; this difference was observed for all journals except *J Applied Ecology*. This difference in the proportion of women invited to review was greatest for older editors and lowest for younger editors; the proportion of women among invited reviewers increased with seniority (age post‐PhD) of female editors but not male editors (for whom the slope was negative, although not statistically significant). Both of these results generalize findings previously reported for *Functional Ecology* (Fox, Burns, & Meyer, 2016a). This gender difference in reviewer recruitment with editor seniority could be caused by differences in professional networks between senior men and women if editors choose reviewers based on personal experience. Or it might result from an effort by more senior women scientists to involve women in the review process, possibly in a conscious effort to promote women in science. Regardless of the cause, these findings suggest a path toward improving the gender balance of reviewers. Journals can emphasize to their editorial boards the intellectual benefits to the field of having diverse reviewers. They should also highlight the observation that male editors and particularly senior male editors tend not to invite as many women and discourage editors from selecting reviewers based entirely on personal experience (which necessarily leads to a bias against the less senior but more diverse population of available reviewers). They can also suggest concrete strategies for identifying more women who would be qualified reviewers, such as using online publication databases or reference sections of papers to identify newly publishing authors. When editors do identify prospective reviewers from personal experience, they can look for postdoctoral scientists working with those established scientists to identify earlier career scientists with relevant expertise to invite as reviewers.

### 4.3 Moving past a gender binary

Research on gender diversity among editors and reviewers is important because it quantifies gender discrepancies and can provide insights into the causes and consequences of inequities in the publishing system. However, for practical reasons, research on gendered outcomes in the publication and grant review process generally impose a gender binary, often based on a person's name (e.g., Cox & Montgomerie, 2019; Débarre et al., 2018; Fox, Burns, Muncy, & Meyer, 2017b; Fox et al., 2018). Yet, nonbinary and transgender scientists are also members of our community (Yoder & Mattheis, 2016); treating gender as binary, and ignoring nonbinary and transgender scientists in our analyses, may send the message that they do not belong or are not part of our science, a message we do not wish to send. Misgendering of individuals also contributes to the excess stress that members of minoritized groups face, which can lead to reduced participation (McLemore, 2015). And, treating gender as binary ignores an important component of gender diversity in scientific publishing, one for which researcher biases and a history of discrimination are especially acute. Future research should consider gender diversity more broadly and inclusively. To that end, journals, professional societies, and funding bodies (such as the US National Science Foundation) should begin collecting data on gender in a way that recognizes nonbinary gender diversity (see Broussard, Warner, & Pope, 2018 and Montague‐Hellen, 2018 for discussions on how to query about gender in surveys).

## 5 CONCLUSIONS

Since 2006, women have earned about half of all doctorates in the biological sciences in the United States (National Science Foundation, 2019). Despite this, women remain much less than half of the population of editors and reviewers of scholarly publications. We explored some of the potential causes and consequences of this pattern, and how gender diversity of editors and reviewers has changed over time, using a dataset from six ecology and evolution journals. Our results suggest a glass that is half full and half empty. One of the encouraging patterns is that the proportion of reviewers and editors who are women has increased consistently over time. By 2015, women were relatively well‐represented on editorial boards (29% of the editors in our dataset) compared with their representation in the reviewer pool (27% in our dataset) and in the pool of last authors of ecology papers (23% in an analysis of papers published from 2010–2015; Fox et al., 2018). On the glass‐half‐empty side, women were underrepresented as reviewers (27% in 2015 in our dataset) compared to the pool of authors (31% women authors across all author positions; Fox et al., 2018) of ecology papers published between 2010 and 2015, but especially compared with the membership of the societies that publish these journals (British Ecological Society and the Ecological Society of America, which were 40% and 37% women, respectively, in the later periods of our database). However, the representation of women in these societies is lower among nonstudents than among students (Martin, 2012), so the under‐representation of women is not as extreme as comparison to society memberships would suggest; for example, women make up 40% of all members of the Society for the Study of Evolution (which publishes *Evolution*), but only 33% of nonstudent members (Débarre et al., 2018). Educating editors on these widespread gender differences in reviewer recruitment, and encouraging editors to use a diversity of approaches (rather than relying primarily on personal experience) to identify prospective reviewers, and especially encouraging editors to identify junior scientists that can be recruited as reviewers, will promote greater equality of participation in the scholarly peer review process.

## CONFLICT OF INTEREST

The authors have no competing interests.

## AUTHOR CONTRIBUTIONS

CWF and JAM collected the data, CWF analyzed the data, CWF and MAD wrote the manuscript, and JAM and DJF commented on the manuscript.

## Data Availability

An anonymized version of the dataset removing personal identifying information (author names and locations) will be published on Dryad.
